# What Racism Has to Do with It: Understanding and Reducing Sexually Transmitted Diseases in Youth of Color

**DOI:** 10.3390/healthcare9060673

**Published:** 2021-06-04

**Authors:** Marie-Claire Boutrin, David R. Williams

**Affiliations:** 1 Department of Biological Sciences, Oakwood University, Huntsville, AL 35896, USA; mcfboutrin@gmail.com; 2 Department of Social and Behavioral Sciences, Harvard T.H. Chan School of Public Health, Boston, MA 02115, USA; 3 Department of African and African American Studies, Harvard University, Cambridge, MA 02138, USA; 4 Department of Psychiatry and Mental Health, University of Cape Town, Groote Schuur Hospital Observatory, Cape Town 7925, South Africa

**Keywords:** STDs, Black youth, youth of color, racism, youth development

## Abstract

Sexually transmitted diseases (STDs) are high in populations of color compared to Whites. High-risk sexual behaviors are widely viewed as the key contributors to the levels of STDs, especially in adolescents and young adults. This article situates the sexual risk behaviors of Black, Indigenous, and other young people of color within the framework of racism. It begins with an overview of racial inequities in common STDs and shows how racism gives rise to several risk factors for high-risk sexual behaviors. These risk factors for STDs identified in prior research are best understood as adaptations to the challenges and constraints faced by youth in socially disadvantaged environments. Both social adversity and the mental health problems that it triggers can lead to risky sexual behaviors. Drawing on findings from prior research with youth of color, this paper describes the needed interventions that can markedly reduce STDs and their risk factors. It also describes needed research on interventions that could contribute to the knowledge and understanding of the adverse conditions fueled by racism that affect youth of color, their health, and their communities.

## 1. Introduction

The American narrative of the racial inferiority of Blacks has long had a sexual dimension, with negative stereotypes of the sexuality of Blacks being an important part of this history [1]. For example, major medical journals in the early 20th century stereotyped Blacks as having excessive sexual desire with promiscuity and a complete lack of morality among Black females and with Black males characterized as having an over-development of their genital organs and an insatiable sexual desire for White women [1]. These stereotypes had consequences on social equality and health. It was stereotypes about the high prevalence of syphilis in a poor Black community and the reluctance of Blacks to seek treatment if they lacked severe symptoms that provided the early rationale for the Tuskegee Syphilis Study in which Black males were deceived and denied treatment for syphilis by U.S. government researchers [1].

There is currently an epidemic of HIV/AIDS and other sexually-transmitted diseases (STDs) in populations of color in the U.S. that receives inadequate attention in health and social policy. This article begins with recent national data on the prevalence of three common STDs, where the prevalence of each is most elevated for the Black (or African American) population. Guided by the framework of racism, it illustrates how STDs in young people of color are best understood in the context of the social and economic marginalization experienced by these populations. We provide an overview of how multiple mechanisms of racism create distinctive demands and challenges that shape the lived experiences of socially disadvantaged youth. We show how behaviors related to STDs are embedded in these larger contextual factors. Using a positive development framework [2], we describe research that identifies strategies that can foster positive youth development and reduce health risks including those linked to STDs. Our research recommendations show how greater alignment of strengths, assets and opportunities for healthy development in youth of color (YOC) and their communities can strengthen competent functioning and improve health and social well-being in these populations.

## 2. Prevalence of STDs in YOC in the U.S.

The Centers for Disease Control and Prevention (CDC) produces detailed annual re-ports on the population prevalence of three STDs—chlamydia, gonorrhea, and syphilis. Chlamydia is the most common bacterial STD in the U.S., and the CDC estimates that there were four million chlamydial infections in 2018. [3]. It is transmitted by vaginal, anal, or oral sex and most persons with chlamydia do not have any symptoms (90% of males and 70–95% of females), so many youth do not perceive a need to be tested for it. However, if untreated, it can cause serious damage to a woman’s reproductive system. It can cause acute and subclinical pelvic inflammatory disease (PID), which can lead to chronic pelvic pain, tubal factor infertility, and potentially fatal ectopic pregnancy. For women with PID, the risk of tubal factor infertility increases from 8% after one episode of chlamydia to as high as 40% after three episodes [4].

Table 1 shows the rates of chlamydia in 2018 for males and females, ages 13 to 24, by race and ethnicity in the U.S. [5]. It also displays the gender ratios and the minority/White ratios for males and females. Table 1 shows substantial variation in the rates of chlamydia by race/ethnicity and sex. For females, compared to Whites, the elevated rates, in order, are for Blacks, American Indians/Alaska Natives, Native Hawaiians and other Pacific Islanders, and Hispanics. A similar pattern is evident among males. The odds ratios for the racial/ethnic minority group compared to Whites, for males and females respectively, are: 6.4 and 4.1 for Blacks, 2.3 and 2.6 for American Indians, 2.1 and 2.5 for Pacific Islanders, and 1.2 and 1.2 for Hispanics. Asians have lower rates than Whites. Furthermore, females have higher rates of chlamydia than males for all groups, with the sex ratios ranging from 2.1 to 4.0, probably reflecting gender differences in access to medical care.

Gonorrhea is also a common bacterial STD. The CDC estimates that there were approximately 1.6 million new gonococcal infections in the United States in 2018, and more than half occur among young people aged 15–24 years old [6]. It is transmitted by sexual contact with the penis, vagina, mouth, or anus of an infected partner and it can also be spread from a mother to her baby during childbirth. Untreated gonorrhea can cause serious health problems for women and men. These include PID and infertility in women and disseminated gonococcal infection (DGI) which can lead to arthritis, tenosynovitis, and/or dermatitis [6].

Table 1 presents the rates for gonorrhea in 2018 for the 13 to 24 year old age group [5]. Across all racial and ethnic groups except Asians, the rate of gonorrhea infections is higher among females than males. Similar to the pattern for chlamydia, for all racial/ethnic groups, except Asians, minority males and females have higher rates of gonorrhea than their White counterparts, with gaps being especially pronounced for Blacks, American Indians, and Pacific Islanders. The elevated odds ratios for racial/ethnic minorities compared to Whites are, for males and females respectively: 11.2 and 7.7 for Blacks, 3.2 and 4.3 for American Indians, 2.5 and 2.2 for Pacific Islanders, and 1.7 and 1.1 for Hispanics. Asians have lower rates than Whites.

Syphilis is also a bacterial STD that can have serious adverse effects on health. It is transmitted from person to person by contact with a syphilitic sore, known as a chancre. This transmission can occur during vaginal, anal, or oral sex. There were 129,813 reported new cases during 2019, and most of them were among gay, bisexual, and other men who have sex with men (MSM) [7]. Pregnant women with syphilis can transmit it to their unborn child. In 2019, there were 1870 cases of congenital syphilis. Some people with untreated syphilis develop tertiary syphilis, which can adversely impact many different organ systems, including the heart and blood vessels, and the brain and nervous system.

Table 1 shows the social patterning of primary and secondary syphilis for individuals aged 13 to 24 in 2018 [5]. Unlike the pattern for chlamydia and gonorrhea, for all racial/ethnic groups, males have higher rates of syphilis than females, and the differences are substantial. The highest overall rate among males is for Blacks, followed by Pacific Islanders, Hispanics, and American Indians, in that order. For females, the highest rate is for Blacks, followed by American Indians, Pacific Islanders, and Hispanics. With the exception of Asians, both males and females belonging to minority racial/ethnic groups tend to have higher rates of syphilis than their White counterparts. Taken together, data from Table 1 show a striking pattern of elevated rates of STDs for both male and female minority youth compared to Whites, with the pattern being especially pronounced for African Americans.

Other data reveal that youth of color are disproportionately affected by HIV. In 2018, males were approximately 79% of all adolescents and young adults aged 13 to 24 years old who were living with diagnosed HIV infection in the U.S. [8]. Among the 30,524 youth aged 13–24 years old with HIV infection, 59% were Black, 24% were Latino, 15% were White, and nearly 2% were Asian. Less than 1% each were Native Hawaiian/other Pacific Islander and American Indian/Alaska Native [8].

### Data Limitations in Reporting of STDs in YOC

It is important to note that the nationally reported data on the prevalence of STDs by race and ethnicity may be inaccurate. These CDC data come from clinic surveillance reports and there is some evidence of differential reporting of STDs to the CDC based on the location of the screening. Specifically, there may be underreporting of cases of STDs at private versus public clinics, and private clinics may be less likely than public ones to report racial/ethnic information along with a confirmed case [9]. A study in one of America’s most populous counties found that racial and ethnic data were reported to the CDC for 95% of cases for public clinics but only for 66% of cases from private clinics [9]. Blacks were three times more likely than Whites to be in public versus private clinics. A national probability survey of 7300 U.S. physicians who cared for patients found that 37% of doctors reported cases of chlamydia, 44% reported cases of gonorrhea and 53% to 57% reported cases of syphilis, HIV, and AIDs [10]. Some 25% to 34% of physicians reported that they instructed their patients to inform the health department about their STD instead of doing so themselves. These biases could artificially inflate observed disparities in the prevalence of infection by race and ethnicity, given that minorities are more likely than Whites to visit public sector reproductive health clinics [11].

Another important limitation is the incompleteness of racial/ethnic status in these officially reported STD rates [12]. For 2018 data for the nation, racial/ethnic status was missing for 29% of chlamydia case reports ranging from 0.1% to 94% by jurisdiction. For gonorrhea, 20% of case reports were missing racial/ethnic data, ranging from 0% to 92% by jurisdiction. Similarly, for primary and secondary syphilis, racial/ethnic status was missing from 4.5% of all case reports, ranging by jurisdiction from 0% to 47%). We do not know the extent to which the patterning of missing data varies across the racial/ethnic categories.

At the same time, population-based surveys document that racial disparities in STD prevalence among adolescents are substantial. For example, a study utilizing data from the National Health and Nutritional Examination Survey (NHANES) found that 1 in every 4 (24%) U.S. females aged 14–19 was infected with at least one of five STDs (HPV, gonorrhea, chlamydia, herpes, and trichomoniasis) tested for in this study [13]. The prevalence was 49% among teens with two or more years of sexual experience and 54% among teens with three or more sex partners. The STD infection rate was 34% among poor teens and 44% for Black teens. A comparable analysis for males was not reported but these data suggest that the prevalence of STDs among adolescent males is also very high.

## 3. Racism and the Constraints and Challenges It Creates for Youth of Color

A growing body of research suggests that there are multiple mechanisms and pathways through which racism leads to pathogenic conditions that facilitate the observed elevated risks of STDs in youth of color (see Table 2).

### 3.1. The Multiple Faces of Racism

In the context of the U.S., racism has been conceptualized as an organized societal system, in which Whites, the dominant racial group, use their power to categorize, rank, and restrict access to valuable social resources and opportunities to racial groups regarded as inferior [14,15]. Accordingly, racism functions as a fundamental driver of racial inequities in health [16,17]. That is, it creates conditions that trigger adverse changes in health, and these changes will persist as long as the basic or fundamental causes remain intact. There are three key mechanisms of racism [15,18]. First, institutional or structural racism refers to racial inequities that are embedded in the routine policies and procedures of social institutions that create unequal outcomes by limiting access to opportunities and rights of the groups deemed inferior. Second, cultural racism encompasses social ideologies that initiate and sustain negative racial images and stereotypes that can reduce support for egalitarian policies, trigger health-damaging psychological responses in stigmatized groups, and facilitate explicit and implicit biases in the larger society that reduce the access of stigmatized groups to desirable resources. Third, differential treatment (discrimination) by both institutions and individuals, in addition to reducing access to opportunity, can be experienced as stressful life experiences that can adversely affect mental and physical health.

### 3.2. Residential Segregation and Socioeconomic Status

Residential racial segregation is one of the most influential institutional (or structural) mechanisms of racism. This term refers to the historic physical separation of the races by enforced residence of Blacks and other stigmatized groups in certain residential contexts so that Whites were protected from residential proximity to them. Research reveals that it reduces access to quality elementary and high school education, employment opportunities, and pathways to socioeconomic mobility [19]. One national study, using rigorous statistical analyses, found that the elimination of segregation would erase Black–White differences in income, education, and unemployment, and reduce racial differences in single motherhood by two-thirds [20]. More recently, Raj Chetty and colleagues [21] have shown, using national data for the U.S., that conditional on parent’s income, Black children have lower incomes than White children and that the driver of these differences in social mobility is the marked differences in resources and opportunities at the neighborhood level.

### 3.3. Residential Segregation, Family Structure, and Parenting

A female-headed household is an aspect of family structure that is linked to the conditions of concentrated poverty created by residential segregation that has important implications for the health and risk behavior profiles of socially disadvantaged youth [22]. Studying the 171 largest cities in America, Robert Sampson [22] shows how lack of access to jobs in segregated neighborhoods produces high male unemployment and underemployment. This, in turn, leads to high rates of out of wedlock births, female-headed households, and the concentration of poverty. His research shows that boys raised in female-headed households receive lower levels of supervision and monitoring, which likely creates greater opportunities to engage in sexual behavior. Importantly, he finds that the association between male unemployment, poverty, and family structure with adolescent risk behavior such as violence is identical in sign and magnitude for Whites and Blacks. That is, higher levels of youth risk behaviors are evident for Blacks compared to Whites, because Blacks are more exposed than their White peers to the underlying social conditions. Thus, racial differences in adolescent risk behavior are driven by racial inequities at the neighborhood level in availability of jobs, family structure, opportunities for marriage, and concentrated poverty [22].

Other research confirms that being raised in a single-parent household is associated with lower levels of parental monitoring and predicts increased risk for multiple outcomes. Children raised in single-parent homes are more likely to become sexually active at a younger age, use illegal drugs, and engage in illegal activities [23]. Research indicates that parental supervision and support is a key mechanism in adolescence and young adulthood, and lower levels of parental monitoring, as observed in some single-parent households, is predictive of a variety of adverse outcomes including substance use and sexual activity, both of which are recognized risk factors for increasing STDs [24,25,26]. Parental monitoring was also found to be a protective and crucial stress-buffering factor, and a promoter of healthier coping strategies to handle distress [27]. Externalizing behaviors such as aggression, risky sexual behavior, and substance use have been reported in adolescents from households with less parental monitoring [27,28]. Parental practices such as involved vigilant parenting—a high level of parental control with affectionate behavior—was shown to foster planful, thoughtful, achievement and future-oriented, alert, attentive, and cautious behavior, and discouraged substance use and high-risk sexual behavior in Black adolescents, with long-term effectiveness [29].

Other research indicates that having residential fathers is crucial for adolescent sexual health. Recent findings from the National Longitudinal Study of Adolescent to Adult Health revealed that adolescents’ perceptions of disapproval from their father concerning their sexual behavior was associated with lower levels of risky sexual behavior, and perceived closeness of Black male adolescents with their father was linked to a decrease in dating violence [30,31].

Thus, the conditions created by segregation that include high levels of exposure to economic adversity and psychosocial stressors, along with the stress of racial discrimination, create family and social environments where levels of parental involvement in teaching about sexual behavior, especially to young men of color, is low. A recent national study of approximately 2000 Black and White 14–17 year olds found that, while White adolescents tend to obtain most of their learning on sexual activity and sex-related topics from parents, Black youth, especially males, acquired most of their sexual learning from the media and peers [32]. Furthermore, sexually explicit material on the internet, printed, and/or in movies was shown to play a primordial role in the sexual development and knowledge of sexual organs, determination of readiness for sex and sexual performance, as well as, sexual behaviors (e.g., no condom use and/or swallowing of ejaculate) in young men of color attracted to the same sex [33]. Learning about sexual matters from parents has been associated with safer sex, more favorable perceptions and attitudes towards condoms, and higher condom self-efficacy [34,35].

### 3.4. Segregation and Stress

Segregation also creates communities characterized by concentrated poverty, poor housing quality, and poor neighborhood physical, social, and chemical conditions that trigger elevated levels of exposure to psychosocial stressors. Because of segregation, communities of color are often concentrated in residential contexts characterized by elevated exposure to chronic and acute stressors and reduced access to a broad range of resources that enhance physical and mental health. The combination of lower economic status and residence in disadvantaged, segregated neighborhoods and exposure to racism leads to higher levels of multiple forms of stress. For example, a study using a probability sample of adult residents in Chicago, a city with higher rates of residential racial segregation, found that compared to Whites, Blacks and U.S.-born Latinos had higher levels of major life events (e.g., death of a child or a life-threatening illness or accident), financial stress (e.g., difficulty making ends meet at the end of the month), relationship stress (e.g., friends and relatives making too many demands), and neighborhood stress (e.g., had home broken into in the past six months) [36].

One consequence of the concentrated poverty of segregated neighborhoods and communities is increased exposure to violence at home or in the community. Violence exposure in the community is associated with lower emotional comfort, family involvement, academic and work performance, and increased individual risk [37]. Exposure to community violence was shown to be associated with several negative outcomes such as psychological distress that persists over time, academic deficiencies mainly due to depressive symptoms, concentration impairments and narrow attention, posttraumatic aggressive symptoms leading to fights and disciplinary actions, lower achievement expectations, greater rates of condomless anal intercourse, marijuana use as a sex drug, and decreased use of anti-retrovirals in HIV-infected youth [27,38,39].

### 3.5. Interpersonal Discrimination as a Stressor

Another mechanism by which racism adversely affects health is via the experience of interpersonal discrimination. Incidents of interpersonal discrimination are an added type of stress faced by youth of color. It can lead to mental trauma, mental health issues, and overall poor health [40]. Significant cross-sectional and prospective associations have been identified between racism-related stress experienced by youth and depression, anxiety, behavior problems, aggression, low self-worth, self-esteem and resilience, and the dysregulation of biological systems leading to poorer physical health, including elevated levels of stress hormones, weight and blood pressure [18,41]. A recent review of longitudinal studies documented a strong association between racial discrimination in childhood and adolescence and increases in behavioral and mental health symptoms [42].

Research also reveals that heavy use of social media and smartphones is adversely associated with the mental health of teens and young adults, as well as with academic performance and sleep [43]. Social media is also a place where youth of color encounter experiences of discrimination that adversely affect their mental health. Research reveals that teens experience relatively high levels of discrimination in online contexts, and these experiences are positively related to depression and anxiety symptoms even after adjustment for demographic factors, a global measure of adolescent stress, and experiences of discrimination in offline contexts [44]. More recently, a study of Black and Latino adolescents found that exposure to traumatic images or videos online (seeing persons from their ethnic group beaten, arrested or detained, or being shot by the police) was linked to higher PTSD and depressive symptoms [45].

### 3.6. The Criminal Justice System, Incarceration, and Stress

Incarceration and other policies within the Criminal Justice System are another example of structural racism that has implications for living conditions that can give rise to risky health behaviors. Rates of incarceration in the U.S. have increased dramatically since the 1970s, with non-White men markedly overrepresented in the prison population. Disparities in surveillance, prosecution, and sentencing are associated with a 10-fold increase in the risk of incarceration for non-Hispanic Black compared to White men in the United States, often reinforced by policies which have differentially criminalized substance abuse and mental illness [46].

Research has documented ways by which incarceration can affect risk behaviors. The duration and level of parental incarceration typically reduces household income and economic standing, therefore reducing the wellbeing of children [47]. This reduction in income may not recover after the parent is released from incarceration, with some 54% of Black youth from low SES backgrounds continuing to occupy low SES backgrounds into adulthood [47]. Low SES and poverty generate financial stress, one of the “serious stressors” that is particularly disastrous when chronic, and is a recognized risk factor for risky sexual behavior and STDs [48].

There is also convincing evidence that youth of color with incarcerated parents develop physical, behavioral, mental health, and school outcome issues including educational difficulties, juvenile incarceration, and substance use [49,50,51,52,53]. Juvenile incarceration and substance use have been described as STD-associated risk factors. Studies have shown that youth of color who are incarcerated tend to have, prior to incarceration, higher rates of STDs and display high-risk behaviors such as substance use, depression, earlier onset of sexual activity, multiple sexual partners, less condom use, substance use during sexual encounters, and that while incarcerated, they tend to affiliate with peers who are delinquent and substance users, continuing their high risk behaviors, even after their release into society [54,55,56].

### 3.7. Sexual Behavior and Coping with Stress

Research indicates that many behaviors, including high-risk sexual behavior, are closely linked to stressors linked to various social determinants of health including low SES, poor housing conditions, discrimination, and undocumented immigration status [57,58,59]. One study shed light on the dynamics of HIV transmission by documenting how sexual and drug-injection networks, combined with higher-order social structures and processes (e.g., residential racial segregation and racialized policing), contribute to elevated HIV risk among Blacks [57,60]. Other evidence indicates that poverty and other structural factors (e.g., high rates of incarceration of Black men and geographically concentrated disadvantage) create a risk environment that is conducive to HIV transmission and other high risk behaviors including sub-optimal engagement in HIV care among low-income Blacks living with HIV [61].

A recent study of 365 Black youth, aged 18 to 24 years old, reported that their biggest concerns were worries about aggressive policing, high levels of community violence, and instability in housing [62]. As predicted by the adaptive calibration framework [2], research finds that youth of color develop stress responses to cope with the severity of the challenges that they are forced to confront in their environment. These youth live with constant threat and fear and manifest high levels of hopelessness, low perceived economic opportunity, and uncertainty about the future [62]. Research finds that, compared to Whites, Blacks and other racial and ethnic minority groups are markedly more pessimistic that they will survive to age 35 [63]. In response to these conditions, many youth live in the moment and view having sexual intercourse as one source of relief from stress [62]. Research has shown that sexual activity such as unprotected penile–vaginal intercourse (PVI) has many mood-enhancing benefits, including increases in relationship and life satisfaction or feelings of love, and can have a protective effect against depression [41,64]. The production of biological factors such as dopamine, oxytocin, and serotonin during PVI largely contributes to the feeling of wellbeing, bonding, closeness, and satisfaction and can facilitate psychological wellbeing and stress-coping mechanisms in youth [41,64]. The use of protection through condoms during PVI was shown to compromise some of these psychological benefits and can be associated with depression and other psychological disorders [41].

Research has also found that negative emotions in youth of color arising from stressors, including discrimination, societal oppression, racism, and violence can lead to depression, antagonism, hopelessness, and a desire to escape adverse situations through “quick fix” coping mechanisms. Quick fix strategies such as sexual behaviors can offer immediate but short-term stress relief, are avoidant coping mechanisms, have a highly addictive potential, and boost high-risk behaviors [29]. In addition, high-risk sexual practices are often unprotected and may arise from a desire for optimum sexual pleasure, peer pressure, spontaneous sexual activity, lack of STD and safe sexual practices awareness, or reproductive coercion [41,65]. These practices contribute to the high rates of STDs in youth of color and are associated with other quick fix coping mechanisms such as drug use [38,66,67,68].

### 3.8. Disparities in Mental Health and Mental Health Care

As noted previously, the multiple facets of racism can adversely affect mental health. A review of research on youth mental health in the U.S. documented that the rate of children and teens going to hospitals due to suicidal thoughts or attempts almost doubled between 2008 and 2015, with the greatest increase among girls [43]. Within this larger context, youth of color stand out with dramatic increases of mental health risks. This pattern is strikingly evident in data on youth suicide. Historically, Blacks have had lower rates of suicide and suicidal behavior than Whites but a growing body of recent research documents markedly rising rates for Black children and youth and a marked narrowing of the Black–White gap in suicide. For example, between 1993 and 2012, national data for the U.S. revealed that suicide rates among elementary school students (aged 5 to 11 years old) declined for Whites, remained stable for Asians and Latinos but doubled for African Americans [69]. This trend of increasing suicide for Blacks has been evident for some time. A study of youth suicide between 1982 and 2001 found that the suicide death rate among Black youth has been increasing faster than that of any other racial/ethnic group [70]. Further analyses using national data on suicide rates for persons age 5 to 17 between 2001 and 2015 revealed that the suicide rate among those younger than 13 years of age was twice as high for Black children compared with White children, and this pattern was evident for both boys and girls [71]. Importantly, this pattern of age-related racial differences in suicide was consistent across the period of the study.

Regarding mental health issues as a contributing factor to risky sexual behavior and STDs, Black adolescents with mental illnesses were shown to engage in more HIV/STD risky behavior than their peers because of poor judgement, limited self-control, decreased problem-solving skills, and the use of sex for intimacy with others or as a coping mechanism for stress relief [72].

We have noted that multiple facets of racism affect the mental, emotional and behavioral health of youth of color, with one of the observed outcomes being disparities in STDs. Another way that racism contributes to this process is through barriers to mental health care encountered by youth of color. Racism in the culture that leads to assumptions and negative stereotypes of youth of color shapes access to care and the quality of care that youth of color receive. Research reveals that patient–provider relationships are adversely affected by provider biases and discrimination and this contributes to mental health cases being untreated, the deterioration of mental health status and the persistence of chronic mental illnesses into adulthood [73,74]. Implicit, unconscious, and underrecognized biases have also been shown to significantly influence differences in treatment decisions, adherence, health outcomes and patient–provider relationships, that result in longer wait times to get an appointment, the receipt of fewer privileges, having shorter consultation times, and fewer questions answered by providers, as well as being addressed in a condescending way [75]. Explicit bias, directly expressed, (believing that Blacks are less educated, less intelligent, prone to risky behaviors, and less likely to be compliant) has also found to be woven into a provider’s diagnostic assessment, treatment plan, or interactions with Black patients [75]. For example, in comparison to their non-Hispanic White peers, Black youth were found to have significantly lower odds of receiving or being referred for treatment for substance abuse, or of getting access to counselling services when they were part of the welfare system [73]. In addition, disparities in mental health care in populations of color may be reinforced by the stigma attached to mental illness, deficiencies in the number of culturally competent mental providers, lack of health insurance, and a general lack of trust of the health care system because of previous negative experiences [76]. Further, the COVID-19 pandemic has disproportionately affected Blacks and other populations of color in the USA, aggravating their vulnerability to mental health issues because of increased experiences of the death of loved ones, grief, loss, experiencing the suffering of loved ones, fear of getting infected by the disease, anxiety, decrease of social support systems, and fear of racial bias and discrimination in disease testing and treatment. As a result, communities of color are likely to experience more trauma, leading to more emotional and physical symptoms such as anxiety, helplessness, nausea, and headache, causing them to seek more stress-relief and soothing outlets [76].

## 4. Opportunities for Developing Interventions Built on the Resources and Strengths of Youth of Color

This section will review evidence suggesting that there are multiple opportunities for positive youth development that can lead to reduced risky sexual behaviors in youth of color (see Table 3).

### 4.1. Addressing the Underlying Social Determinants of Health Behaviors

An emerging body of scientific evidence suggests that addressing the underlying social determinants of health (economic status, housing, employment opportunities) must be an essential part of a comprehensive approach to reducing risky sexual behaviors among low-income racial and ethnic minority populations. A shift from a narrow focus on behavioral change approaches to an emphasis on the upstream social determinants can play an important role in comprehensive efforts to reduce racial inequities in STDs. Much of the best evidence comes from studies that have targeted risky behaviors linked to HIV/AIDS. These studies reveal that interventions that strengthen women’s economic well-being, housing stability, and gender empowerment are associated with improved psychological well-being, economic productivity, and reduced HIV risk [77]. For example, an intervention with a group of drug-using women who were involved in prostitution taught the women strategies of HIV risk reduction and also how to earn income by making, marketing, and selling jewelry. Three months later, these women reported lower levels of receiving drugs or money for sex, fewer sex trade partners, and less money spent on drug use [78]. Similarly, an intervention with Black women who used crack and had risky sexual behaviors taught them strategies to enhance their power and control over health-related behaviors but also addressed their personal independence and economic resources. This intervention documented improvements in housing and employment, as well as reductions in drug use and high-risk sexual behaviors compared to women in a standard HIV prevention intervention group [79]. Similarly, a randomized controlled trial (RCT) found that providing stable housing for homeless and unstably housed persons with HIV led to reductions in their risk behaviors, increased access to care, and increased adherence to their medication regimen [80].

The Mpowerment project is the name of an intervention targeted at men who have sex with men that has been shown, in multiple communities, to be effective in reducing unsafe sexual behaviors [81,82]. The key to this intervention is a multi-leveled approach that combines reducing HIV-related risk behaviors at the individual level with community-level and individual-level capacity building and empowerment. There are several key features of this project [81]. First, the intervention is targeted at the community level and not just at men who engage in high risk behaviors. Second, based on early focus groups with participants, the intervention ties HIV risk reduction with addressing other challenges faced by the young men such as gay bashing, discrimination, and conflicts over gay rights. Accordingly, the intervention seeks to build self esteem, strengthen the men’s social network, and enhance their enjoyment of social interactions. Relatedly, there is also an effort to strengthen the young adults’ pride and celebration of their sexuality that seeks to present safer sex materials in a graphic and attractive way. Third, at the community level, the intervention strives to strengthen friendships and networks of social support. It created positive settings for social interactions (as opposed to bars and cruising settings) and seeks to foster norms that encourage caring for one another and enhancing individual growth. Fourth, the intervention also uses peers as the key sources for the transmission of information about risk behaviors. It recruits, trains, and encourages young men to be active in encouraging others to have safer sex. Fifth, the empowerment emphasis of this intervention encourages young men to be active in identifying and deploying solutions to their problems. The intervention also involves a Community Advisory Board that links the leaders of local organizations and their resources to address the young men’s social needs.

Other evidence confirms that interventions that improve underlying living conditions can enhance the development of protective sexual behaviors in Blacks. Research indicates that improving education and affordable housing can reduce HIV and AIDS incidence rates because low levels of education and unstable housing have been found to decrease social stability and thereby increase HIV risk behaviors such as risky sex and drug use [83,84]. A meta-analysis of 70 studies examining the effects of residential segregation and Whites’ attitudes towards Blacks on sexual risk behavior prevention interventions showed that both Whites’ positive attitudes toward African Americans and lower levels of residential segregation were associated with improved condom use [85]. Importantly, these two structural-level factors accounted for the variance in condom use effect sizes above intervention-level characteristics and community-level education and poverty levels [85]. Similarly, a qualitative study found that among Black males experiencing unemployment, hunger, and housing instability, addressing these underlying social determinants with multi-level interventions would decrease risky sexual behaviors [86].

A key message from these studies is that efforts to reduce STD risk behaviors should be comprehensive and include addressing explicit attention to improve other desirable outcomes in the low-economic status contexts where many youth of color reside. Sexual risk factors should optimally be addressed in the context of broader public health and socioeconomic initiatives and interventions in these communities. Thus, the risky sexual behaviors of youth of color should be viewed as catalysts for the implementation of health and social programs that seek to enhance a broader range of positive outcomes for youth of color and their communities. Comprehensive interventions focused on HIV prevention and sex education can increase awareness, enable the practice of safer sexual behaviors and resilience, and act as preventative strategies against high-risk behaviors associated with life stressors that are disproportionately affecting these communities. As a result, children, adolescents, and young adults from those communities could grow up with an increased awareness of safe sexual behaviors and resources to sustain safe sexual practices. For example, a pilot HIV prevention intervention that sought to improve knowledge and attitudes about HIV/AIDS and reduce risky sexual behaviors in Afro-Caribbean female teenagers, also focused on facilitating better mother–daughter communication. It demonstrated that when these teens felt the need for sexual activity, the parental support fostered through the intervention enabled them to use values, knowledge, and skills to make healthier choices and reduce risky sexual behaviors [87].

Another intervention for gay and bisexual young men of color living with HIV named Brothers Building Brothers by Breaking Barriers was found to be promising because of its holistic, culturally specific approach. The initiative did not only focus on the usual targets (e.g., medical adherence, appointment attendance, sexual health), but also aimed to build resilience, competence, confidence, and connectedness by addressing psychosocial factors such as identity, self-reflection, family skills, and community involvement, as well as a range of healthy behaviors and efforts to create a supportive social network [88]. Similarly, since substance use has been shown to be linked to higher numbers of sexual partners and higher rates of STDs in youth of color, interventions targeting substance abuse may have a positive impact on risky sexual behaviors among youth of color [89]. A study reviewing six effective national HIV/STD and pregnancy reduction intervention programs among youth of color revealed that future programs should incorporate more emphasis on avoidance or limited substance use, increased parental involvement, and the integration of cultural teaching [90].

### 4.2. Building on Other Psychosocial Assets

Research also suggests that there are a number of psychosocial resources that can be incorporated into successful interventions to reduce high-risk sexual behaviors. Appealing to the status, independence, and maturity of young adults is one strategy. This approach is based on the recognition that the removal of sexual protection such as condoms by young men of color can have a larger symbolic value. It can be motivated by a desire to be unconditionally loved, to have a sense of belonging, to build a legacy through fatherhood, and to become responsible for the welfare of self and others [65]. These motivations are consistent with the construct of masculine respectability whereby men desire to uphold social order and support high family functioning [65]. It follows that interventions that promote responsible and safe sexual behaviors as a part of masculine respectability have the potential to foster positive youth development in young men of color, especially when combined with community-level programs supporting their family- and legacy-building desires.

Pseudo-mature behaviors (adult-like behaviors) such as sexual activity can also fill a need for acceptance and belonging for some youth of color who grow up in socioeconomically challenged environments with feelings of rejection and little self-esteem and confidence. Thus, interventions that focus on enhancing peer-acceptance can boost self-worth, confidence, a sense of belonging and eventually competence in these youth of color, and gear them towards normative healthy sexual behaviors at later stages of life. A study that examined the association between adolescent peer acceptance and sexual outcomes in adolescence and young adulthood documented that despite its association with early sex initiation, peer acceptance in adolescence was associated with healthy sexual behaviors in adulthood [91]. These findings highlight the potential of the promotion of peer-acceptance to enhance the practice of safe sexual behaviors in youth of color.

Research also indicates that enhancing racial pride for socially stigmatized racial populations can lead to better behaviors, including sexual behavior [18,29,92]. That is, building racial pride can be an explicit strategy for mitigating some of the negative effects of the devaluing of stigmatized groups that is deeply embedded in the pervasive negative ideology about marginalized racial groups that is part of the larger culture. An RCT of Black and Latino 16- to 18-year-old males who were being released from jail compared the standard single session of jail-based, discharge planning to an eight session, 30-h educational intervention that began in jail and continued after the teenagers were released. The curriculum addressed drugs, sexual relationships, risk factor reduction, and also explicitly devoted time to building racial pride in the youth. A year later, the teens that had received the comprehensive intervention had spent fewer days in jail, were more likely to have attended school and found work, and had reduced odds of substance abuse [93]. Similarly, an RCT with African American girls aged 14 to 18 focused on building racial pride, gender empowerment, and solidarity with the black community into the four 4-h sessions. This intervention also enhanced HIV-prevention behaviors and skills, with increased condom use evident at 6 months and 12 months after the intervention [94,95].

Religious involvement is another potential resource. Research indicates that in addition to family and school connectedness, religious involvement can help with coping with stress and its sequelae through supportive relationships founded on common values and beliefs with other youth and adults [96]. Religious involvement can play a role in development from childhood to young adulthood that can steer some youth towards peers with fewer problem behaviors, help define identity formation, especially religious identity, and reduce the negative effects of stress on psychological wellbeing [97]. Some studies find that religious involvement is associated with lower depression, higher self-esteem, life satisfaction, thriving and coping, and delayed sexual initiation among Black youth [96,98,99]. Greater religious involvement was also associated with decreased delinquency, male LGBT identity, substance use, sexual risks, higher school bonding, and student–teacher connectedness [100].

Additionally, churches can offer platforms for sexual health education and STD interventions. A study of an STD intervention in North Carolina demonstrated that Black churches can be instrumental in youth of color interventions [101]. This intervention promoted the knowledge and skills about sexual health issues, built awareness about HIV/AIDS, improved decision-making skills and communication between youth and their parents by enabling parents to learn and practice skills that fostered good communication with their children on sexual risk reduction. Although the intervention was not primarily faith-based, the Black churches facilitated the coming together of networks of families, youth, and religious leaders from the community in trusted and supportive environments for participants with opportunities to “end the silence” and create discussions about sexual health issues in safe and credible places in the Black community [101]. Similarly, a pilot study of a STD prevention program in 12 Black churches in Michigan demonstrated the potential reach of church-based interventions. Some 1382 individuals participated in this program which offered HIV testing, distribution of condoms, training and engagement of leaders and youth in sexual health education and STD prevention, cross-gender communication about sex, sexuality and relationships, and the discussion of risky behaviors regarding HIV/AIDS transmission [102]. A recent systematic review of the role of faith-based organizations in HIV prevention programs in the Deep South also found that Black church-based programs can reduce racial disparities in HIV infection [103].

### 4.3. Health Care Opportunities

In general, adolescents and youth of color face healthcare barriers including limited access to healthcare and out-of-pocket costs that drive disparities in receiving health education, disease prevention, and diagnosis and treatment [104]. Multiple solutions are needed to address this challenge. First, policies are needed to ensure that all youth of color have access to preventive and treatment services. Second, youth of color must be encouraged and enabled to pursue careers in the health professions, including mental health care, to build a cadre of health professionals who can understand, relate, empathize, and advocate for the health of all youth of color. A study documenting that greater household income was associated with an increased risk of major depressive disorder in African American male adolescents highlights the need of health services at every level of SES in at least some populations of color [105].

## 5. Directions and Recommendations for Future Research

Future research on interventions in youth of color should seek to maximize their capacities and strengths and align them to promote the development of positive outcomes including low-risk sexual behaviors. There is a need for the deployment and rigorous evaluation of comprehensive interventions in youth of color that seek to maximize personal capacities and strengths including low-risk sexual behaviors, as well as community and societal characteristics that will reduce discrimination, inequalities, prejudices, bias, police brutality, destructive social media and many other factors that adversely affect the mental health of youth of color. Federal and state efforts to reinforce the creation and implementation of policies and programs promoting physical, mental, emotional and financial health in families and youth of color are urgently needed.

Future research should also provide a more comprehensive intersectional understanding of the social distribution of STDs. The data presented in Table 1 focused only on age, race/ethnicity, and sex. We do not know how these patterns vary by SES (income and education). Failure to stratify racial health data by the social factors that contribute to them can mis-specify social risks and even reinforce harmful racial stereotypes [106]. More generally, Ross and Fernandez-Esquer [107] recommend that a broad range of social variables should be considered when reporting and seeking to understand racial and ethnic differences in the prevalence of STDs. These include socioeconomic status, religious affiliation, country of origin, family structure, place, discrimination, and differential access to medical care.

Another research priority is to assess the extent to which there may be optimal windows for implementing particular interventions. This would require more explicit consideration of the age of the youth of color and their stage in puberty. For example, a cross-sectional study involving 411 mainly Black adolescents reported that early pubertal timing is a beneficial time for positive reinforcement practice by parents to generate positive behavior modification in early maturing males [108]. At that stage of development, early maturing males would display an increased reward-oriented behavior tendency and a decreased ability for emotion regulation compared to on-time or late-maturing peers because of the hormone-activated remodeling of the dopaminergic pathway in their brain [108]. Furthermore, a recent meta-analysis of 54 studies and a systematic review of 25 studies revealed several associations between various types of early life adversity and sexual risk behaviors in later years, suggesting a crucial role for early-life interventions in communities of color to combat the range of early life adversities faced by children [109]. This review documented that threat-related early life adversity was associated with accelerated pubertal timing, which was linked to several health problems (including increased risky behaviors, delinquency, substance abuse problems, depression, anxiety disorders, and physical health problems such as cardiovascular disease, polycystic ovarian syndrome and testicular cancer). Further, varying types of early life adversity (e.g., threat, deprivation, SES) were associated with accelerated cortical thinning, a marker for brain development, which is linked to attention-deficit/hyperactivity disorder and both internalizing and externalizing psychopathology [109]. Considering that the majority of these physical and mental health dysfunctions are notably observed in low income populations and populations of color, longitudinal studies involving early interventions in children facing adversity should be done to evaluate the extent to which the effects of these interventions vary with age at the time of their implementation and to identify the conditions under which these interventions are more or less impactful for the reduction of accelerated development, and risk factors for physical and mental health problems.

Future interventions should also take into consideration lessons learned from previous initiatives, including successful strategies that led to long lasting positive outcomes such as healing, wellbeing, and resilience. Research is also needed to identify the conditions under which the large racial/ethnic disparities in healthcare systems, for both physical and mental health care, can best be minimized and eliminated. Sneed and colleagues [76] recently described a multifaceted intervention in the state of Michigan that aimed to address the notable disparities in access to mental health care in the Black community, especially as this population was being disproportionately adversely affected by COVID-19 and was experiencing high levels of grief, loss, and fear. The intervention was culturally sensitive, trauma-informed, and used evidence-informed models for processing stress and trauma, such as the mindfulness-based stress reduction and the Community Resiliency Model (teaching of wellness skills to decrease physical sensations of stress and trauma). The intervention was initiated following the acknowledgement by state and local leaders that there were significant health disparities that required immediate responses to address them. Second, multisector task forces were put in place to address issues of racial bias related to testing, diagnosis, and treatment. Furthermore, videoconferences and webinars sponsored by community groups such as Black faith communities and academic institutions promoted the dissemination of disease-related information. Further, federal funding coupled with local public mental health providers created virtual crisis centers to support mental health and substance abuse screening and dismantle barriers to treatment engagement in Black communities. Teams made up of local community leaders, academic clinical psychologists, and public health professionals conducted virtual support groups and sessions for those who were not in crisis but needed a safe platform to discuss their fears and stressors [76]. This intervention demonstrates the importance and feasibility of using societal, community, and individual strategies when developing and implementing culturally sensitive, tailored, and comprehensive interventions. Interventions like these need to be rigorously evaluated so that there is better understanding of what are the key aspects of comprehensive interventions that are critical to their success.

Similarly, other studies have shown that socio-structural barriers must be removed and positive identity, relationships, cognition/emotions, sense of community, self-worth, acceptance, sense of belonging, and other aspects of a holistic approach must be implemented to develop resilience, competence, and confidence in youth of color, rather than simply focusing on STD treatment, prevention, and clinical success, [68,88,110]. One of the ways to dismantle those barriers could be to use culturally appropriate venues such as barbershops for reaching youth of color. A qualitative systematic review involving cross-sectional studies showed that barbershop-based interventions can be successfully used in the prevention, education, and screening of Black males for various health topics [111]. After the implementation of adequate training, barbershops have been successfully used as platforms for sex education, prevention, and the screening and dissemination of health publications to youth of color [111]. Furthermore, the needs of youth of color with mental illnesses must be carefully considered, and barriers such as transportation, potential breaches of confidentiality, and time would need to be addressed. Along with these, adequate mental health treatments should be incorporated to provide a holistic intervention [72].

To address the earlier noted challenge of lower supervision of adolescents raised in single parent homes, programs and policies creating stable employment opportunities for Black parents should be evaluated for their potential to improve parental time and supervision. Research on military enlistment shows that increased employment opportunities for males has positive effects on family formation and stability. Military enlistment is a program that provides disadvantaged Black men increased education, higher earnings, and greater occupational mobility than their civilian peers [112]. In addition, military benefits include resources that support maintaining a family: family housing, day care centers, and school-age activity centers. Research reveals that active duty military service promotes marriage over cohabitation, increases the likelihood of first marriage, and leads to greater stability of marriage and all of these effects were greater for Blacks than for Whites [112,113,114]. These data suggest that economic resources could markedly reduce racial disparities in marriage, which, in turn, could enhance the family context for reducing risky adolescent behavior. Evaluating this hypothesis with other employment initiatives should be a priority in future research.

Future research should also examine the role that social media can play in the delivery of interventions targeting the sexual behaviors and health of youth of color. Research indicates that youth of color are heavily influenced by and reliant on media sources for their sexual education, determination of time of sexual initiation, readiness for sex, sexual performance, and risky sexual behaviors [32,33]. In addition, social media is instrumental in teenagers’ sexual health education through the use of social networking and text messaging, and the development of interactive web-based STD management interventions [115]. A cross-sectional study found that young people exposed to sexual health messages on social media were nearly three times more likely to have used contraception or a condom during their last sexual intercourse while other sources of information such as parents, schools, and traditional media did not show significant associations [116]. Furthermore, a qualitative study involving youth of color reported the potential of text messaging as an intervention medium for safer sexual behaviors (e.g., safe sex negotiation and discussion tool) with potential partners before engaging in sexual activities [117]. In addition, social media also offered the ability to obtain information, communicate with healthcare professionals, and secure help without having to face the stigma that is often associated with STDs and mental health issues [118]. Some available platforms are CureTogether.com, a website that allows patients to share information on sensitive symptoms and discuss the efficacy of their treatments, and PatientsLikeMe.com, which enables patients to use social networking to learn about disease symptoms and treatment options, and also to report health benefits such as reduced risky sexual behavior, which would be valuable information for youth of color [115]. Further, social media can facilitate access to youth of color who are vulnerable and hard to reach and could be greatly instrumental in attempts to decrease health disparities. As an example, StartUpHealth.com, a program that included over 8000 health and wellness innovators, allowed the dissemination of entrepreneur-driven ideas to patients and providers as well as health opportunities to underserved and Medicaid communities [115]. Therefore, further research on social media and related phone applications use should be strongly encouraged to promote the development and implementation of new comprehensive sexual health programs that would provide access to information, treatment, and discussions on STDs and related topics to communities that are hard to reach, underserved, vulnerable, and that stigmatize STDs.

Research is also needed to explore the extent to which values affirmation interventions, possibly combined with other educational interventions, could be effective in reducing STD risk behavior in youth of color. Values affirmation interventions are brief exercises that require youth to select their most important value and write a paragraph on why that value was selected and why it was important to the individual. The goal of the exercise is to improve the participant’s sense of personal adequacy and self-worth. These interventions in students have led to higher grades, large reductions in the racial achievement gap, a greater belief in the student’s ability to succeed in school, and an enhanced ability to deal with stress [119]. They have also been associated with better mental health, stress management, performance, self-confidence, and well-being [120,121]. These psychological interventions appear to reduce at the least some of the deleterious effects of cultural racism (stereotype threat and internalized racism) among students and lead to improved health behaviors in studies of teens and young adults [119]. Future research should assess the extent to which values affirmation interventions can have positive effects on reducing adolescent sexual risk behaviors.

Research is also needed to better understand the determinants of variation in risky sexual behavior in youth of color. All youth of color are not sexually active [122]. Our current understanding of the determinants of virginity is limited. One study found that virginity (absence of PVI) was linked to BMI, physical attractiveness, educational attainment, anxiety, self-esteem, religion, two-parent household residence, having antisocial friends, sexual abuse, and cigarette use [122]. More research is needed to better understand the prevalence and the determinants of virginity across various populations of youth of color and to identify the nature and extent of its relationship with other indicators of positive youth development.

## 6. Conclusions

In conclusion, there is an urgent need to use the evidence presented in this review to design and implement needed interventions to address the large disparities in STDs evident among youth of color. This review highlighted several promising interventions that could be replicated and scaled for future health campaigns, even as we recognize the necessity of additional research that would shed light on the need to better identify the optimal programs and interventions that would reduce high-risk sexual behaviors in youth of color. The available evidence highlights the importance of situating the prevalence of STDs in youth of color in the larger social, economic, cultural, and psychological contexts in which they are embedded. A high prevalence of risky sexual behaviors emerge in racially stigmatized, disadvantaged contexts and there is an urgent need to accelerate initiatives to dismantle the structures of racism that created and sustain these social inequities and identify countervailing interventions that can enable youth to transform their lives, find healing from past traumas, and build legacies of success in their physical health, psychological well-being, socioeconomic status, and families. Finally, there is an urgent need to identify the relative importance and contribution of the various protective factors that we have identified, the optimal timing and sequencing for implementing these interventions, and the optimal combination of strategies that are likely to have the greatest impact.

## Figures and Tables

**Table 1:** Sexually-transmitted disease rates (per 100,000) for the 13–24 age group, by sex, race and ethnicity, U.S., 2018.

			Sex	Minority/White	
	Rate per 100,000		Ratio	Ratio	
Race/Ethnicity	Female	Male	Female: Male	Female	Male
			Chlamydia		
American Indian/Alaska Native	3835.4	1029.9	3.7	2.6	2.3
Asian	642.1	204.3	3.1	0.4	0.5
Black ^1^	6075.6	2858.3	2.1	4.1	6.4
Hispanic/Latino	1807.7	554.5	3.2	1.2	1.2
Native Hawaiian ^2^	3732.3	931.8	4.0	2.5	2.1
Non-Hispanic White	1488.3	443.8	3.4	- *	- *
			Gonorrhea		
American Indian/Alaska Native	924	422	2.2	4.3	3.2
Asian	56.6	83	0.7	0.3	0.6
Black ^1^	1665	1483.6	1.1	7.7	11.2
Hispanic/Latino	243.5	222.8	1.1	1.1	1.7
Native Hawaiian ^2^	472.3	325.3	1.5	2.2	2.5
Non-Hispanic White	215.4	132.6	1.6	- *	- *
			Syphilis		
American Indian/Alaska Native	12.7	26.3	0.5	4.2	2.6
Asian	1	12.8	0.1	0.3	1.3
Black ^1^	19.6	66.8	0.3	6.5	6.5
Hispanic/Latino	4.9	26.4	0.2	1.6	2.6
Native Hawaiian ^2^	10.1	38.5	0.3	3.4	3.8
Non-Hispanic White	3	10.2	0.3	- *	- *

*: Ratios are calculated using the Non-Hispanic White group as the reference. ^1^ Black = Black or African American ^2^ Native Hawaiian = Native Hawaiian and other Pacific Islander. Calculations from the Center for Disease Control, AtlasPlus tool, https://gis.cdc.gov/grasp/nchhstpatlas/tables.html Accessed on 29 December 2020. [5].

**Table 2:** Summary of pathways by which racism increases risks for youth of color.

Mechanisms by which racism can affect stress and behaviors Residential segregation (structural racism): Reduces access to opportunities and leads to reduced income, education and employment; Results in concentrated poverty that leads to father absence; Is linked to lower levels of parental supervision, especially of boys; Increases exposure to violence, and elevated levels and greater clustering of other chronic and acute stressors; Is linked to high levels of aggressive and racialized policing; Leads to emotional distress, substance abuse, deviant behavior, and risky sexual behaviors. Discrimination (interpersonal racism): Interpersonal discrimination in social contexts is an added source of stress; Youth of color also experience discrimination in online contexts; Exposure to images of police brutality online are also stressful; All of these stressors have adverse impacts on youth mental health. Criminal justice system policies and incarceration (structural racism): Triggers disparities in surveillance, prosecution, sentencing; Increases duration and levels of parental incarceration; Leads to lower socioeconomic status and financial distress; Is linked to adverse physical, behavioral, emotional and school outcomes; Increases youth incarceration; Increases stress, mental health problems and risky health behaviors. Exposure to stressors and disadvantaged contexts (triggered by racism) leads to risky behaviors and mental health problems: Stress and adversity among youth is linked to high-risk sexual behavior; High levels of threat and fear lead to high hopelessness and uncertainty about the future; Because of low perceived opportunity, many youth live in the moment and may view sex as a source of relief from stress; Youth of color with mental, emotional, and behavioral problems are more likely to engage in risky sexual behavior. Cultural racism contributes to reduced access to mental health care: The mental health of youth of color is worsening over time; Suicide rates of blacks aged 5 to 13 doubled between 1990 and 2015 and are twice as high as those of their white peers; Low SES creates barriers in access to care; Implicit and explicit provider biases lead to poorer care: longer wait times, shorter consultations, fewer questions answered by providers; These biases also lead to misdiagnosis and lower levels of referral for appropriate care.

**Table 3:** Summary of opportunities for developing interventions built on the resources and strengths of YOC.

Interventions that address the underlying social determinants of health: Interventions focused narrowly on behavioral change have limited impact; Addressing underlying social determinants (economic status, jobs, education, housing) can lead to reductions in risk behavior; Community-level interventions that promote capacity building and empowerment of youth also reduce risky behavior; Comprehensive, culturally specific approaches that address STD risks within the broader contexts of the lives of youth (e.g., healthy behaviors in general, needed social programs and skills, enhancing supportive social networks) show promise; Interventions that incorporate the promotion of psychosocial resources, such as peer-acceptance and racial pride are also promising; Faith-based interventions and those that build on psychosocial assets linked to religious participation and religious communities can also play a role in STD prevention and reduce risky sexual behaviors. Interventions that increase access to care and the quality of care: Youth of color face barriers in access to healthcare and the quality of care; Policy interventions are needed to enhance access across the continuum of care from prevention through treatment; Interventions are also needed to increase career opportunities for youth of color in all aspects of health care delivery, including mental health care.

## Data Availability

A publicly available dataset was analyzed for the data presented in Table 1 of this article. These data are available from the Centers for Disease Control and Prevention in AtlasPlus. They are available online at: https://gis.cdc.gov/grasp/nchhstpatlas/tables.html, accessed date 4 June 2021.
